# A case of a stuck mesh in the rectum after pelvic surgery

**DOI:** 10.1002/deo2.286

**Published:** 2023-09-15

**Authors:** Makoto Eizuka, Yosuke Toya, Risaburo Akasaka, Shun Yamada, Tomofumi Oizumi, Satoshi Kasugai, Shunichi Yanai, Yoshihiko Sugimura, Takayuki Matsumoto

**Affiliations:** ^1^ Department of Internal Medicine Division of Gastroenterology and Hepatology School of Medicine Iwate Medical University Iwate Japan; ^2^ Department of Surgery Morioka Red Cross Hospital Morioka Iwate Japan

**Keywords:** granulation tissue, magnifying endoscopy, mesh, pelvic surgery, rectal foreign body

## Abstract

A 67‐year‐old woman was referred to our hospital for further evaluation of a positive fecal occult blood test. Colonoscopy revealed an elevated rectal lesion (10 mm in size) with a central depression. A rod‐like object was noted in the center of the lesion. Magnifying endoscopy with narrow‐band imaging showed obscure surface structures and dilated vessels. Magnifying endoscopy with crystal violet staining showed that the pit pattern had disappeared. These endoscopic findings suggested that the lesion was comprised of granulation tissue. A detailed medical history revealed that she had undergone a total hysterectomy with mesh placement for bladder prolapse. We reasoned that the mesh used during pelvic surgery might have penetrated the rectum. She underwent subsequent surgery to remove the mesh. Although most foreign bodies in the rectum are swallowed or self‐inserted, pelvic surgery is another source of foreign bodies in the rectum.

## INTRODUCTION

Although rectal foreign bodies (RFBs) are rarely encountered in clinical practice, RFBs can cause bleeding, mucosal ulceration, and perforation.^1^, ^2^, ^3^, ^4^ Transanal removal of RFBs is performed in many cases, but some may require urgent surgery.^1^, ^2^, ^3^, ^4^ Most RFBs are diagnosed by medical history and radiographs, but there are several reports of RFB diagnosis by endoscopy.^1^, ^2^, ^3^, ^4^ Most RFBs are swallowed or self‐inserted secondary to accidental events, mental retardation, sexual preference, or suicide attempts; iatrogenic RFBs are very rare.^5^ Herein we report an iatrogenic RFB diagnosed by medical history and unique endoscopic findings.

## CASE REPORT

**FIGURE 1:**
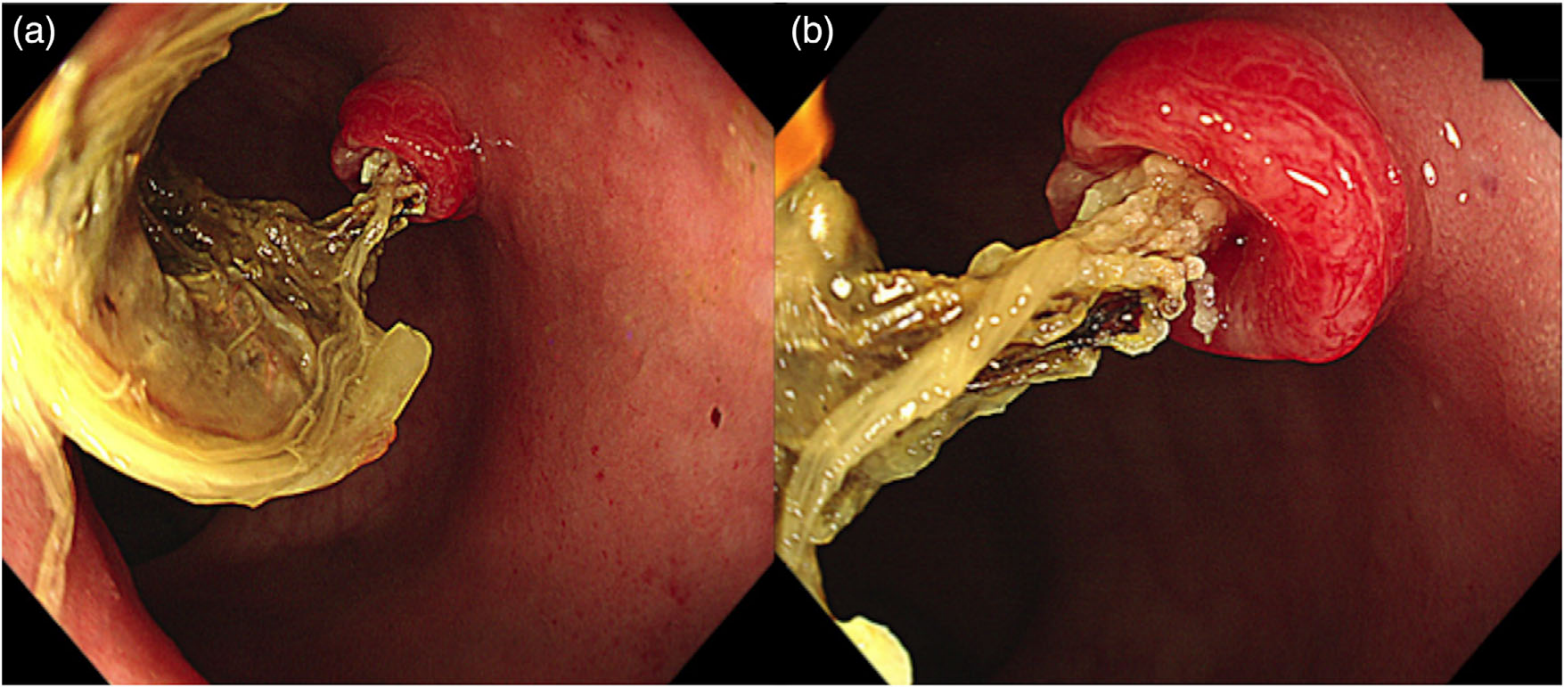
(a, b) Colonoscopy revealed an elevated lesion with a rod‐like object in the center.

**FIGURE 2:**
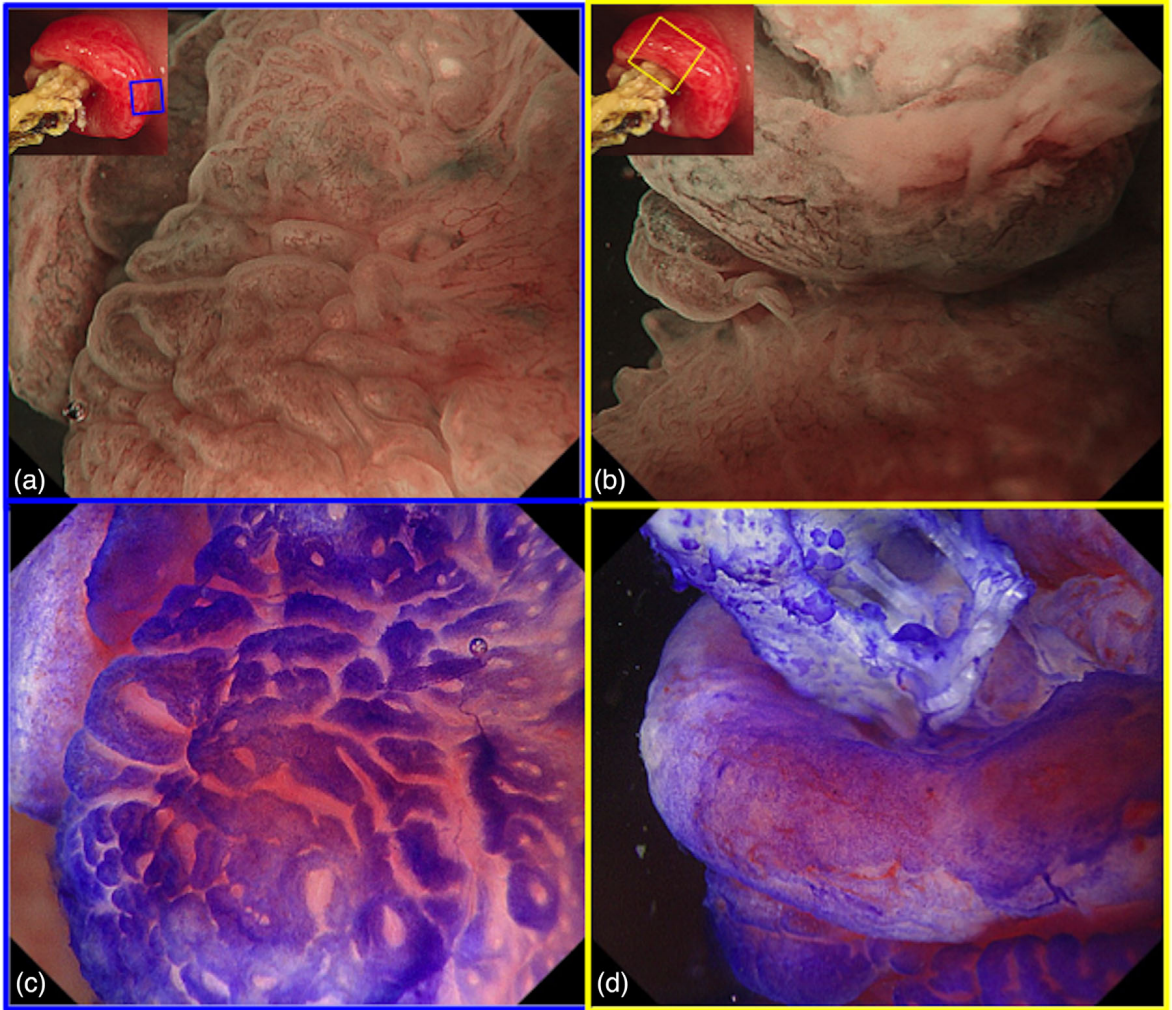
(a, b) White light image of the elevated lesion with a rod‐like object in the center. A magnified endoscopic image is highlighted with a blue or yellow frame. Magnifying endoscopy with narrow‐band imaging showed obscure surface structures and dilated vessels. (c, d.) Magnifying endoscopy with crystal violet staining showed that the pit pattern had disappeared.

A 67‐year‐old woman was referred to our hospital for further evaluation of a positive fecal occult blood test. The physical examination and laboratory data were normal. A colonoscopy revealed an elevated lesion (10 mm in size) with a central depression in the anterior wall of the lower part of the rectum. A rod‐like and hard object was observed in the center of the lesion (Figure 1a,b). Magnifying endoscopy with narrow‐band imaging showed obscure surface structures and dilated vessels at the margins of the elevation (Figure 2a,b). Magnifying endoscopy with crystal violet staining revealed that the pit pattern had disappeared (Figure 2c,d). These endoscopic findings suggested that the protruding lesion was comprised of granulation tissue. The patient had been free from routine enema use.

**FIGURE 3:**
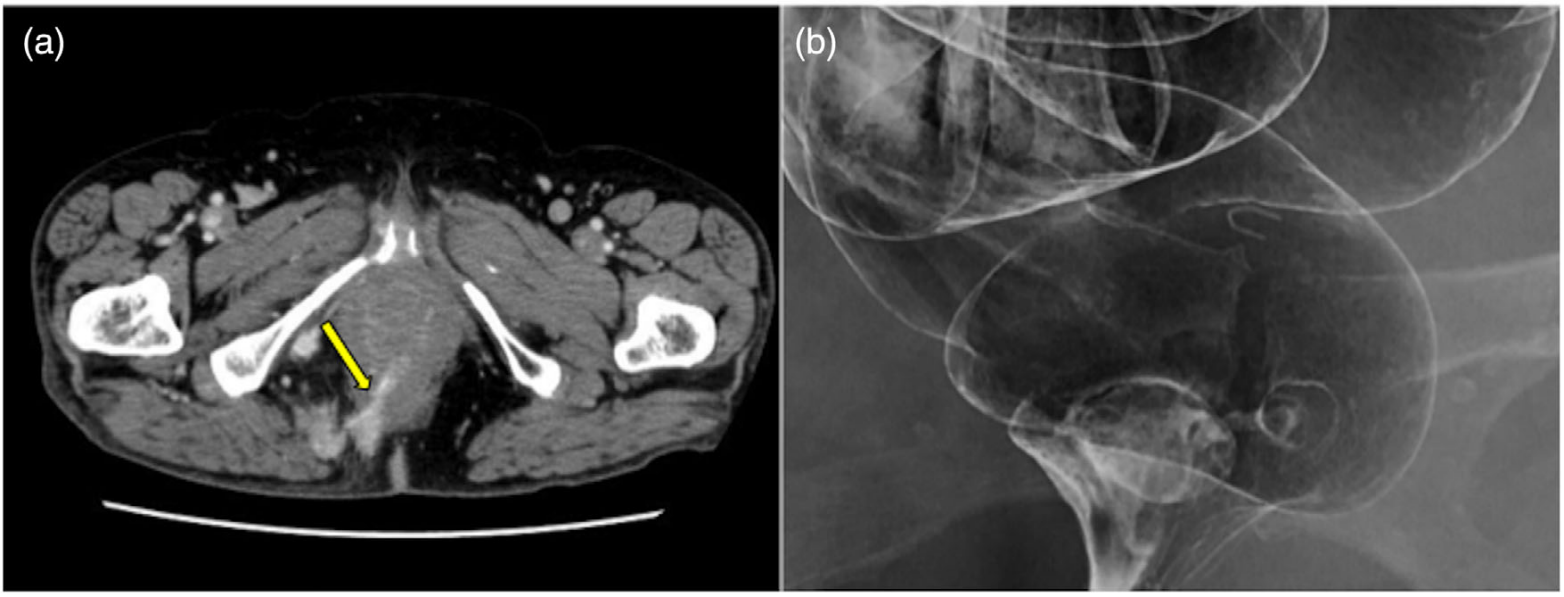
(a) Computed tomography showed a linear, high‐density area puncturing the vulva and rectum (yellow arrow). (b) Under barium enema examination to check for intestinal leakage or a fistula, there was not any leakage of contrast material through the lesion.

We attempted to grasp and remove the rod‐like object with biopsy forceps (Radial Jaw 4; Boston Scientific), but the object was firmly implanted in the center of the lesion. Computed tomography showed a high‐density, linear material that had migrated through the right vulva to penetrate the rectum (Figure 3a). Under barium enema examination, there was not any leakage of contrast material through the lesion (Figure 3b).

A detailed medical history revealed that she had undergone a total hysterectomy for uterine prolapse and placement of mesh for bladder prolapse at 53 and 66 years of age, respectively. Based on her medical history and endoscopic and radiographic findings, we diagnosed this case as an RFB, and the RFB was presumed to be the penetrating mesh used in pelvic surgery. Considering the risk of bleeding and abscess formation due to RFB, we chose surgical removal of the material. The surgical procedure consisted of transanal removal and suture closure, during which it was noted that one of the six supporting legs applied to fix the mesh had penetrated the rectum. The patient was discharged to home without complications after surgery.

## DISCUSSION

RFBs can be complicated and difficult to treat depending on the type and size of the foreign body. There are several patterns in the pathogenesis of RFBs. Zhang et al.^5^ analyzed 291 cases with RFBs, which were classified as follows: swallowed RFB (*n* = 199 [68.4%]); self‐inserted RFB (*n* = 87 [29.9%]); and iatrogenic (*n* = 5 [1.7%]). Of the five iatrogenic RFBs, four occurred following a procedure for prolapse and hemorrhoids in which staples were left in the rectum, and one case involved a uterine contraceptive device retained in the rectum. An online search in PubMed of reports published between 2012 and 2022 identified seven cases of iatrogenic RFBs, including the report by Zhang et al. (Table 1).^5^, ^6^, ^7^ Five patients had intrarectal foreign bodies; three patients, including our case, involved RFBs that penetrated the rectum from outside of the gastrointestinal tract. Iatrogenic RFBs are relatively rare, but cases of extraintestinal penetration are even rarer. Our case is the first report of an RFB in which mesh used during pelvic surgery penetrated the rectum.

**TABLE 1:** Iatrogenic rectal foreign body (RFB) cases.

Reference	Age (years)	Gender	Symptom	Category of RFB	Treatment that caused RFB	Treatment
Zhang et al. (Five cases) [5]	56.6 ± 5.5	Male Two cases Female Three cases	Rectal bleeding Three cases	PPH staple Four cases Intrauterine device One case	Hemorrhoids Four cases To prevent pregnancy One case	Transanal removal Three cases
Kohli et al. [6]	34	Male	Abdominal pain and bleeding	Plastic catheter remnants	Colon hydrotherapy	Endoscopic removal
Shin et al. [7]	39	Female	Abdominal pain	Surgical sponge	Hysterectomy	Lower anterior resection
Present case	67	Female	None	Mesh	Tension‐free vaginal tape procedure	Operation

Abbreviations: PPH, procedure for prolapse and hemorrhoids; RFB, rectal foreign body.

In the present case, the RFB resulted from pelvic surgery for bladder prolapse. A tension‐free vaginal tape (TVT) procedure was performed to stabilize the pelvic organs.^8^, ^9^, ^10^ The tension‐free vaginal tape procedure restores urethral support at the level of the pubourethral ligaments by placing a polypropylene mesh or tape sling at the mid‐urethra as opposed to the bladder neck.^8^ Normally, two meshes are implanted on the anterior and posterior walls of the uterus to stabilize the pelvic organs in the correct position, but in this case, one mesh was implanted on the posterior bladder wall because the patient had undergone a total hysterectomy. Therefore, six additional support legs were added to the mesh for fixation, one of which is thought to have perforated into the rectum. We believe that residual RFB poses a risk of bleeding and abscess formation. To the best of our knowledge, this is the first reported case in which mesh used in a tension‐free vaginal tape procedure penetrated into the rectum. Our experience suggests that an RFB resulting from pelvic surgery should be considered if a firmly fixed object is found in the granulation tissue of the rectum.

In conclusion, we have reported a case of an iatrogenic RFB diagnosed by medical history and unique endoscopic findings. Our experience suggests that although rare, pelvic surgery can be the cause of an iatrogenic RFB.

## CONFLICT OF INTEREST STATEMENT

Takayuki Matsumoto is a responsible and executive JGES member for *DEN Open*. The other authors declare no conflict of interest.
